# Molecular plant immunity against biotrophic, hemibiotrophic, and necrotrophic fungi

**DOI:** 10.1042/EBC20210073

**Published:** 2022-09-30

**Authors:** Carl L. McCombe, Julian R. Greenwood, Peter S. Solomon, Simon J. Williams

**Affiliations:** Plant Sciences Division, Research School of Biology, The Australian National University, Canberra, ACT, Australia

**Keywords:** biotroph, necrotroph, plant immunity

## Abstract

Pathogenic fungi use diverse infection strategies to obtain nutrients from plants. Biotrophic fungi feed only on living plant tissue, whereas necrotrophic fungi kill host cells to extract nutrients. To prevent disease, plants need to distinguish between pathogens with different life cycles, as a successful defense against a biotroph, which often involves programmed cell-death around the site of infection, is not an appropriate response to some necrotrophs. Plants utilize a vast collection of extracellular and intracellular receptors to detect the signatures of pathogen attack. In turn, pathogens are under strong selection to mask or avoid certain receptor responses while enhancing or manipulating other receptor responses to promote virulence. In this review, we focus on the plant receptors involved in resistance responses to fungal pathogens and highlight, with examples, how the infection strategy of fungal pathogens can determine if recognition responses are effective at preventing disease.

## Introduction

Crop-infecting fungi are estimated to be responsible for an annual pre-harvest yield-loss of ∼20% worldwide [1]. The infection strategies used by fungi to obtain plant nutrients can be grouped into three broad categories, biotrophic, hemibiotrophic, and necrotrophic. Biotrophic pathogens extract nutrients from living cells, necrotrophic pathogens kill plant cells to access nutrients, and hemibiotrophs initially extract nutrients from living tissue before switching to a necrotrophic phase.

Plants rely on an innate immune system that is activated by receptor proteins at the cell surface and within the cell [2,3]. Extracellular pathogen recognition is controlled by plasma-membrane embedded receptors, while intracellular recognition often utilizes cytosolic nucleotide binding (NB) leucine-rich repeat (LRR) receptors (NLRs). Receptors are responsible for sensing molecules characteristic of pathogen infection, including microbe-associated molecular patterns (MAMPs), damage-associated molecular patterns (DAMPs), phytocytokines and pathogen-derived virulence molecules, known as effectors (Box 1). Effectors are secreted by adapted pathogens to promote virulence and are utilized by biotrophic, hemibiotrophic, and necrotrophic fungal pathogens to aid in colonization of host plants [4]. The responses activated by plant receptors can include the production of reactive oxygen species (ROS) and anti-microbial compounds, the influx of Ca^2+^ into the cytosol, immune signaling via mitogen-activated protein kinase (MAPK) cascades, transcriptional reprogramming, and a hypersensitive response (HR) leading to localized plant cell death [5,6].

In this review, we summarize recent findings concerning pathogen recognition via plant receptors, with a focus on their capacity to activate appropriate defense responses against biotrophic, hemibiotrophic, and necrotrophic pathogens.

Box 1 **Definitions for the types of molecules that are characteristic of infection and can be sensed by plant receptor proteins to activate defense responses**

**Table:** 

Term	Definition
MAMP	Microbe-associated molecular patterns are compounds derived from molecules required for microbial survival. MAMPs do not actively promote infection, examples include chitin oligomers from fungi and flagellin peptides from bacteria.
DAMP	Damage-associated molecular patterns are plant molecules that are released by cellular damage resulting from pathogen infection.
Phytocytokine	Plant peptides produced in the cytosol and secreted into the apoplast to stimulate plant immunity.
Effector	A secreted molecule, typically a small protein, that functions to promote infection.

## Cell-surface recognition

**Figure 1:**
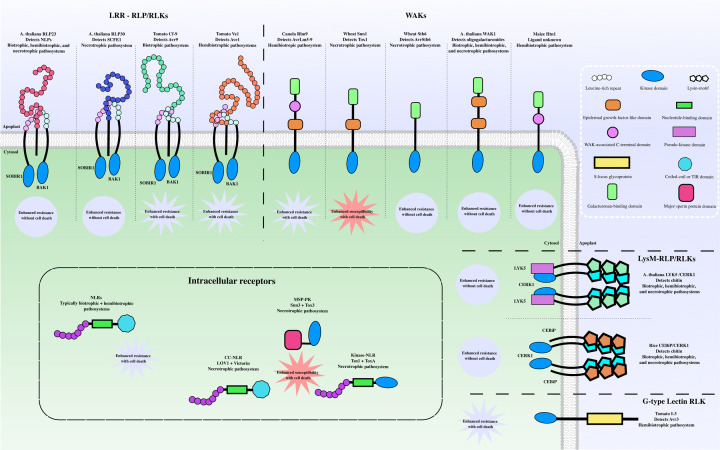
Extracellular and intracellular receptor proteins can promote disease resistance or susceptibility to fungal pathogens Cell-surface receptors are embedded in the plasma-membrane while intracellular receptors are localized to the cytosol. The cell-surface receptors are grouped into subfamilies based on their extracellular domain/s. Lysin-motif receptor-like proteins and receptor-like kinases (LysM-RLP/RLKs) can detect chitin oligomers released from the fungal cell wall to activate defense responses that promote disease resistance against biotrophic, hemibiotrophic, and necrotrophic fungi without leading to plant cell death. Leucine-rich repeat (LRR) receptors can detect secreted proteins (effectors) from pathogenic fungi and mediate downstream immune signaling by forming receptor complexes with other LRR-RLKs (SOBIR1 and BAK1). Notably, the LRR-RLP/RLKs that detect effectors from necrotrophic fungi and promote disease resistance do not lead to plant cell death. Likewise, wall-associated kinase (WAK) receptor proteins activate responses that typically promote disease resistance. However, Snn1 from wheat detects a necrotrophic effector (Tox1) and triggers a cell-death response that supports the growth of the invading necrotroph. Inside the cell, intracellular receptors detect effectors and typically promote disease resistance by initiating a localized cell-death response that restricts the growth of biotrophic and hemibiotrophic fungi. However, multiple necrotrophic fungi use effectors to manipulate intracellular receptors into activating cell death, ultimately promoting disease susceptibility. Only the receptors discussed in this review are depicted in the figure.

Most cell-surface pathogen-recognition receptors are categorized as either receptor-like proteins (RLPs) or receptor-like kinases (RLKs), depending on the absence or presence of an intracellular kinase domain. Both receptor classes have a membrane-embedded domain and an extracellular ligand-binding domain. RLPs and RLKs can be further classified into subfamilies based on their extracellular domain (ECD). Identified ECDs on plant pathogen recognition receptors include lysin motif (LysM), leucine-rich repeat (LRR), lectin (lec), epidermal growth factor (EGF)-like, galacturonan-binding wall-associated-kinase (GubWAK) and WAK-associated C-terminal domain [7,8] (Figure 1). Cell-surface receptors are essential components to many cellular and developmental functions, and provide the first opportunity for detection of pathogen attack. They are therefore subject to manipulation by plant pathogens to facilitate infection and prevent appropriate resistance responses.

### LysM receptors

LysM-RLP/RLKs are essential for microorganism perception and the activation of various plant responses facilitating defense and symbiosis. The ECDs of LysM receptors typically bind to polysaccharides, including chitin oligomers cleaved from the cell wall of invading fungi. Chitin elicitor receptor kinase1 (CERK1) is a LysM-RLK crucial for chitin-induced defense responses and is present in both monocot and dicot plants [9,10]. In rice, CERK1 is recruited by the chitin elicitor binding protein (CEBiP), a LysM-RLP, following homodimerization of CEBiP promoted by binding to long-chain chitin oligomers [11]. The CERK1 and CEBiP complex activates immune signaling pathways and ultimately promotes fungal disease resistance [11] (Figure 1 and Table 1). Rice CERK1 is also implicated in symbiosis with arbuscular mycorrhizal fungi [12,13] and can detect short-chain chitin oligomers produced by mycorrhizal fungi when in complex MYR1 (another LysM-RLK) [14,15]. A recent discovery demonstrated that MYR1/CERK1 complex formation reduces the chitin-induced immune response by inhibiting the recruitment of CERK1 by CEBiP [16], emphasising the complex nature of cell surface perception and signaling.

*Arabidopsis thaliana* has three LysM-RLP homologs of CEBiP, but none of these are required for general chitin-induced immune responses [17]. In *A. thaliana*, two LysM-RLKs with pseudo-kinase domains (LYK4 and LYK5), in addition to CERK1, are indispensable for the typical immune responses following chitin perception [9,18] (Figure 1 and Table 1). Like CEBiP, LYK4/LYK5 interact with CERK1 to form hetero-oligomers and mediate downstream immune signaling following chitin binding [10,18]. It is believed that the oligomerization of *At*LYK4/LYK5 and CEBiP with CERK1 facilitates CERK1 homodimerization and the subsequent phosphorylation of CERK1 that is required for effective downstream signaling [19–21]. The kinase domain of CERK1 potentiates immune signaling by phosphorylating target proteins, including various receptor-like cytoplasmic kinases (RLCKs) [22–27]. In addition to typical intracellular immune responses (ROS production, Ca^2+^ influx, MAPK signaling, transcriptional changes), recent work demonstrated that chitin perception dependent on CERK1 induces stomatal closure, likely inhibiting the entry of certain foliar pathogens [28,29]. For further details concerning CERK1 mediated defense and the methods fungi use to suppress chitin-triggered immunity, we refer readers to a recent review [19].

By recognizing highly conserved fungal cell wall components, LysM-RLPs/RLKs can inhibit the growth of a diverse range of pathogenic fungi, including biotrophs, hemibiotrophs, and necrotrophs. The fact that a single LysM-RLK can both promote the establishment of symbiotic fungi and restrict the growth of pathogenic fungi implies a complex signaling system which probably requires additional receptors and/or co-receptors to differentiate friend from foe.

### LRR receptors

LRR ECDs commonly bind to peptides or proteins (although a well-studied exception is the detection of brassinosteroids (BRs) (reviewed in [30])). Like the LysM-RLP/RLK heteromeric receptor complexes, LRR-RLP/RLKs interact with each other to perceive ligands and potentiate intracellular signaling. To identify LRR-RLK receptor complexes, Smakowska-Luzan et al. used high-throughput assays investigating pair-wise interactions between 200 *A. thaliana* LRR-RLKs [31]. A key finding from the interaction network was that LRR-RLKs with small ECDs (<12 leucine rich repeats) typically interact with LRR-RLKs with large ECDs (>12 repeats) [31]. The concept that small LRR-RLKs are more likely to act as co-receptors for multiple receptor complexes is demonstrated by the well-characterized BAK1, a small LRR-RLK (4 repeats) that forms receptor complexes with multiple large LRR-RLKs, including those required for the perception of BRs (BRI1, 25 repeats) and peptides from bacterial flagellin (FLS2, 28 repeats) [32–36]. Immune signaling via BAK1 and FLS2 also requires the malectin-like receptor kinase FERONIA, which acts as a scaffold that can be regulated by plant RAPID ALKALINIZATION FACTOR propeptides to inhibit plant immune responses [37].

Most LRR-RLP/RLKs that perceive fungal-specific molecules detect effector proteins. *Fulvia fulva* (syn. *Cladosporium fulvum)* is a biotrophic pathogen responsible for leaf mold in tomato. The first effector-detecting LRR-RLP gene identified was Cf-9 (28 repeats) from tomato, that detects the *F. fulva* effector Avr9 [38]. Subsequent work identified more LRR-RLPs responsible for detecting *F. fulva* effectors, in all cases, the receptors lack intracellular kinase domains typically used for signal transduction [39–42]. It was therefore assumed that the effector-detecting receptors form complexes with RLKs to initiate immune signaling. SOBIR1, a small LRR-RLK (5 repeats), was the first Cf-9 interacting receptor identified [43]. Additional research determined that BAK1 and SERK1 (both small LRR-RLKs) are recruited to the SOBIR1/Cf receptor complex following ligand binding and are required for immune activation [44]. The Cf receptors trigger a local HR that restricts biotrophic pathogen growth [45] (Figure 1 and Table 1). HR activating LRR-RLPs are also effective at preventing the growth of certain hemibiotrophic pathogens. The Ave1 effector from *Verticillium, Fusarium* and *Colletotrichum spp.* is recognized by the LRR-RLP Ve1 (38 repeats) in tomato [46,47]. Like the Cf receptors, Ve1 requires SOBIR1 [43] and BAK1 [48] for complete immune activation (Figure 1 and Table 1). LRR-RLPs are also important for disease resistance against necrotrophic pathogens; however in this case the activation of plant immunity does not result in a HR (Figure 1). The LRR-RLP RLP30 (21 repeats) from *A. thaliana* detects a small (16–22 kDa) secreted protein from the necrotrophic pathogen *Sclerotinia sclerotiorum*, activating immune responses and decreasing fungal growth without inducing plant cell-death [49]. Likewise, RLP23 (27 repeats) detects a small peptide from Nep1-like proteins (NLPs) encoded by diverse microbes to activate plant immunity without a HR [50,51] (Figure 1 and Table 1). NLPs can also act as cytotoxic effectors that interact with plasma-membrane embedded sphingolipids and an extracellular plant LRR protein to cause plant cell death, this is believed to promote the virulence of certain necrotrophic and hemibiotrophic pathogens [52–55]. However, NLPs are also encoded by biotrophic pathogens and many are not cytotoxic, suggesting that they likely have another unknown function [55]. By detecting NLPs without inducing a HR, RLP23 increases disease resistance against various biotrophic, hemibiotrophic, and necrotrophic pathogens [50,51,56].

As illustrated in Figure 1, RLP23, RLP30, Ve1, and the Cf-9 receptors all rely on similar RLK co-receptors (SOBIR1 and BAK1) for immune signaling despite differences in the outcome of the immune response [49,51]. The signaling output from LRR-RLP/RLKs is often controlled by the intracellular domain (ICD) and can be uncoupled from the ECD [57]. For example, Wu et al*.* recently reported that swapping the ECD of Cf-9 with the ECD of EFR (an LRR-RLK that does not activate a HR), leads to a HR when the chimeric protein is expressed in tobacco and exposed to the ligand detected by EFR [58]. The exact mechanism by which the ICDs of RLP/RLKs fine-tune immune signaling is currently unknown. Potentially the different responses may be achieved by the different receptor complexes influencing the phosphorylation of the ICD of BAK1 and/or SOBIR1 which in turn affects downstream signaling. Perraki et al. demonstrated that BAK1 has phosphosites that are required for immune responses activated by FLS2 but are not necessary for BRI1 signaling [59]. Future studies characterizing the phosphorylation of BAK1 and/or SOBIR1 in different receptor complexes may uncover how the receptors effective against biotrophs and hemibiotrophs signal for a HR, while the receptors detecting effectors from necrotrophs signal for immune activation without cell death.

The presented examples demonstrate that LRR receptor complexes have the capacity to increase disease resistance against a broad range of fungal pathogens by detecting effectors. Interestingly, the detection of biotrophic/hemibiotrophic effectors typically activates a localized HR preventing biotrophic growth whereas the detection of necrotrophic effectors stimulates the immune system without causing plant cell death that could promote necrotrophic infection [45,46,49,51].

### Lectin domain receptors

Receptors with lectin (carbohydrate binding) domains (Lec-RLKs) are more commonly associated with developmental process rather than defense. Lec-RLKs can be divided into three classes: L-type, C-type and G-type. Only G-type Lec-RLKs are known to detect molecules of fungal origin, for a detailed overview of all types of Lec-RLKs and their potential ligands we refer readers to recent reviews [60,61]. The ECD of G-type Lec-RLKs includes a G-lectin domain, plasminogen-apple-nematode (PAN) motif, and a *S-*locus glycoprotein (SLG) domain. G-type Lec-RLKs are best known for their role in self-incompatibility (SI) systems [62–64]. To prevent self-fertilization, pollen and pistil cells recognize ‘self’ using a secreted small cysteine-rich protein (SCR) and extracellular SLG-domain containing receptor capable of binding to the SCR [65,66]. SCR-binding can trigger homodimerization of the receptor, likely causing transphosphorylation of the ICDs that initiates downstream signaling [66,67]. Studies in field poppy, reviewed in [68], have demonstrated that SI activation can result in ROS production, increased cytosolic [Ca^2+^], irreversible oxidation of plant proteins, cytosolic acidification, and cell death [69–73].

The I-3 protein from tomato is currently the only G-type Lec-RLK known to recognize a fungal molecule [74]. I-3 detects the Avr3 effector from the hemibiotroph *Fusarium oxysporum f. sp. lycopersici* and inhibits pathogen growth (Table 1). Transient expression of Avr3 in I-3 containing tomato or co-expression of Avr3 and I-3 in *Nicotiana benthamiana* does not lead to a HR [74]. However, it was recently demonstrated that infiltration of purified recombinant Avr3 protein into I-3 tomato results in an I-3-dependent cell-death response [75]. Whether I-3 mediated immunity and plant cell death depends on the same signaling components as SI activation remains to be determined. Avr3 is a small cysteine-rich protein, analogous to the SCRs involved in SI, suggesting that Avr3 may bind directly to the I-3 SLG domain [76]. While not sufficient to rule out a direct interaction, previous yeast-two-hybrid experiments were unable to detect binding of Avr3 to the I-3 ectodomain [74]. Avr1, which is recognized by the LRR-RLP I [77], bears structural similarity to Avr3 [75] and is able to inhibit Avr3 resistance responses [78]. The mechanism of Avr3 mediated resistance suppression by Avr1 is unknown, but in-light of the structural similarity it is tempting to speculate that Avr1 interferes with Avr3 mediated recognition by binding to I-3 or a common ‘guarded’ host target. Given the ability of *S-*locus glycoproteins to detect small cysteine-rich proteins and activate plant cell death, it is likely that future research will identify more effector detecting G-type Lec-RLKs involved in promoting plant immunity against biotrophic and hemibiotrophic pathogens.

### WAK receptors

Wall-associated kinases (WAKs) are characterized by a galacturonan-binding GubWAK ECD and often also have an extracellular EGF-like domain. There are 22 members of the WAK and WAK-like gene family in Arabidopsis [79], but these families have expanded in other plant species with 99 members in *Gossypium* *hirsutum* (cotton) [80], 91 members in barley [81], 125 in rice [82] and 555 in wheat [83]. The expansion of WAKs in both monocot and dicot species and the presence of unique structural families within species suggests that WAKs may have evolved following the divergence of monocots and dicots and during speciation. WAK1 from *A. thaliana* was first described to associate with the plant cell wall [84], specifically via pectin [85]. In addition, a recombinantly produced fragment of the WAK1 ectodomain was able to interact with cell wall extracted polygalacturonic acid, oligogalacturonides and pectin [86]. WAK1 was then shown to activate immunity by recognising plant-derived oligogalacturonides [87,88], and recent evidence demonstrates that WAKs can also detect fungal effectors [89–91]. Rlm9 from canola is a WAK immune receptor that perceives the AvrLm5-9 effector from the hemibiotrophic fungus *Leptosphaeria maculans*, the causal agent of blackleg disease [90]. Recognition of AvrLm5-9 by Rlm9 results in a HR and inhibits infection, however whether other immune responses are activated, how Rlm-9 potentiates immune signals, or if Rlm-9 requires co-receptors is unknown [90]. Rlm4 and Rlm7 are allelic variants of Rlm9 and detect the *L. maculans* effectors AvrLm4 and AvrLm4-7 to activate a HR and prevent further infection [91]. AvrLm4-7 and AvrLm5-9 are structurally similar [92], and AvrLm4-7 can inhibit AvrLm5-9 mediated resistance [93]. Like the Avr1 and Avr3 effectors mentioned previously, the mode of inhibition is unknown, but a common means of recognition evasion by pathogens could come from the selection of structurally similar effector proteins that are able to negate established forms of pathogen detection.

A WAK from wheat, Stb6, recognizes the AvrStb6 effector from the necrotrophic pathogen *Zymoseptoria tritici* [89]. Stb6 lacks an EGF-like domain, suggesting that this domain is not critical for the activation of immune responses by WAKs [89]. As opposed to Rlm9, Stb6 does not induce a HR following effector recognition [89]. The Tox1 effector from the necrotroph *Parastagonospora nodorum* is also recognized by a wheat WAK, Snn1 [94]. In contrast to all effector detecting receptors discussed above, the recognition of Tox1 by Snn1 promotes disease through a model known as effector-triggered susceptibility [94–96]. Snn1 activation results in typical immune responses, including the production of ROS, the expression of defense-related genes, and plant-cell death [95]. Both Rlm9 and Snn1 activation leads to plant cell death. However, due to different infection strategies, Snn1 enhances disease-susceptibility in the wheat-*P. nodorum* pathosystem, whereas Rlm9 promotes disease-resistance in the canola-*L. maculans* pathosystem (Figure 1 and Table 1). An exciting avenue for future research could be aiming to engineer Stb6, a WAK that enhances immunity against a necrotrophic pathogen without inducing a HR, to detect Tox1 by swapping ECDs with Snn1. Proof-of-concept domain-swapping experiments using WAK1 and the LRR-RLK EFR have demonstrated that WAK1 immune signaling by the ICD can be uncoupled from ligand-binding by the ECD [87].

Several other WAKs involved in fungal-disease resistance have been described, but their means of pathogen perception and ability to confer resistance require further investigation (Figure 1). Agriculturally important alleles of the maize WAK *ZmWAK-RLK1* are able to delay the progression of northern corn leaf blight (NCLB), but not completely halt the disease [97,98]. A resistance allele of *ZmWAK-RLK1* has been reported to modify the expression of genes involved in jasmonic acid (JA) and ethylene defense pathways, lignin synthesis, and cell wall maintenance, with most of these genes differentially expressed before infection occurs [99]. Unlike the previous WAK examples, alleles of ZmWAK-RLK1 may therefore function independently of the recognition of pathogen derived components to confer resistance.

WAKs are fast emerging as important receptor proteins capable of promoting disease resistance against biotrophic, hemibiotrophic, and necrotrophic pathogens (Figure 1 and Table 1). Similar to LRR receptors, the WAKs that detect effector proteins can activate pathways that lead to cell death or to enhanced immunity without cell death. Whether the response reduces or promotes infection depends on the lifestyle of the pathogen. The expansion of the WAK and WAK-like gene family in plants suggests that this family of genes is under strong selection for roles in defense responses, much like the expanded intracellular nucleotide-binding, LRR receptor (NLR) family (discussed below). Unlike NLRs however, WAKs seem to maintain diverse roles in cell maintenance and development [100], as well as in abiotic [101] and biotic stress responses.

## Intracellular recognition

NLRs are cytosolic proteins that activate immune responses following the detection of an effector. Typically, NLRs are composed of a C-terminal LRR domain, central NB domain, and variable N-terminal domain. The two major classes of NLR are CC-NLRs that have a coiled-coil N-terminal domain and TIR-NLRs that have a toll/interleukin-1 receptor N-terminal domain. In general, the CC and TIR domains are responsible for signalling and the NB and LRR domains regulate NLR activation. The activation of an NLR by a fungal effector typically results in a HR that prevents the growth of biotrophic and hemibiotrophic pathogens [102]. They are the most crucial defense receptor against many adapted pathogens and have been studied and reviewed extensively. For a detailed understanding of how NLRs perceive effectors, and the subsequent immune signaling pathways see [103–106].

Interestingly, necrotrophic pathogens can co-opt NLR effector recognition to promote cell death and enhance disease. LOV1 is an *A. thaliana* CC-NLR that confers susceptibility to the necrotrophic fungus *Cochliobolus victoriae* (Table 1) [107]. LOV1 guards a plant thioredoxin TRX-h5 to detect ribosomally produced cyclic hexapeptides (victorin) secreted by *C. victoriae* during infection, leading to cell death and plant susceptibility [108,109]. Victorin binds directly to the active site of TRX-h5, and in the absence of LOV1 inhibits plant defense [108]. The high conservation of LOV1 across *A. thaliana* accessions suggests that it plays an important role in plant immunity, likely mediating resistance against a biotrophic or hemibiotrophic pathogen by detecting an effector that interferes with TRX-h5 [110]. Victorin-induced susceptibility to *C. victoriae* is also controlled by a CC-NLR in common bean and the gene responsible for susceptibility in oats co-segregates with a resistance gene to the biotrophic pathogen *Puccinia coronata* [111,112]. Collectively, the victorin research indicates that necrotrophic pathogens can promote infection by secreting effectors that mimic recognised effectors from biotrophic or hemibiotrophic pathosystems to activate a HR. An analogous example involves the protein effector ToxA from necrotrophic pathogens *Pyrenophora tritici-repentis*, *Parastagonospora nodorum*, and *Bipolaris sorokiniana.* ToxA is recognized by the intracellular wheat NLR receptor Tsn1, a non-canonical NLR with a kinase domain at the N-terminus (Table 1) [113]. The ToxA protein, in the absence of the pathogen causes plant cell-death in wheat lines carrying Tsn1 [114]. In the context of these necrotrophic pathogens, ToxA is a major determinant in promoting disease in wheat that possess Tsn1 [115–118]. Snn3 is another wheat disease susceptibility gene that encodes an intracellular protein with a kinase domain [119]. The *P. nodorum* effector Tox3 is detected by Snn3, which results in the upregulation of defense-related transcripts, indicating that Snn3 likely functions in plant defense but is being manipulated by Tox3 to promote infection [120]. Unlike previously characterized pathogen-detecting receptors, Snn3 has a C-terminal major sperm protein (MSP) domain [119]. It is predicted that the MSP domain is responsible for effector perception and the kinase domain then activates defense responses, including the plant cell-death that promotes the necrotrophic growth of *P. nodorum*. Genes with MSP and protein kinase domains are specific to monocot plants and significant diversity exists in the sequences of the MSP domains, including the presence of uncharacterized integrated domains [119]. It is tempting to speculate that future research efforts will identify more receptors in monocot plants that use an MSP domain to detect effector proteins and activate defense responses. For further discussion on the various receptors hijacked by necrotrophic pathogens see [121].

To defend against biotrophic and hemibiotrophic pathogens intracellular effector-detecting plant receptors often activate immune signaling that culminates in a HR, restricting the growth of pathogens that require living plant tissue. However, the examples presented here demonstrate how necrotrophic pathogens can manipulate intracellular receptors to promote plant-cell death and enable nutrient acquisition.

## Receptor cross-talk

We have divided this review into sections based on the structure of the immune receptors. However, regardless of structure, most receptors activate similar, overlapping pathways and recent advances have demonstrated that diverse receptors work co-operatively to promote plant immunity. BAK1, the common LRR-RLP/RLK co-receptor, phosphorylates CERK1 following activation, which promotes rapid activation of CERK1 following chitin perception [122]. A cotton WAK positively regulates resistance to wilt-diseases by promoting the heterodimerization of the LysM-RLKs LYK5 and CERK1 [123]. Likewise, WAK1 from tomato acts to promote apoplastic immunity activated by an LRR-RLK [124].

Extracellular receptors can also enhance the immune responses activated by intracellular receptors, and vice-versa. The Cf-4 LRR receptor complex requires a downstream CC-NLR to activate a HR and restrict the growth of the biotrophic invader [125,126]. Furthermore, two recent publications demonstrated that NLR activation results in the up-regulation of extracellular receptor signaling components, and the HR activated by NLRs requires the activation of extracellular receptors [127,128]. For an in-depth discussion on the cross-over between immunity activated by intracellular and extracellular receptors, we refer readers to recent reviews [129–131].

**Table 1:** A summary of the receptors used by plants to detect invading fungi and the outcome of receptor activation as discussed in this review and depicted in Figure 1

Plant receptor and species	Identified co-receptor/s	Molecule detected	Infection strategy of associated pathogen/s	Plant cell death	Outcome (enhanced disease resistance or susceptibility)
LysM-RLP/RLKs					
LYK5 – *A. thaliana*	CERK1	Chitin oligomers	All	No	Resistance
CEBiP – Rice	CERK1	Chitin oligomers	All	No	Resistance
LRR-RLP/RLKs					
RLP30 – *A. thaliana*	SOBIR1/BAK1	A small secreted protein	Necrotroph	No	Resistance
RLP23 – *A. thaliana*	SOBIR1/BAK1	NLPs	All	No	Resistance
Ve1 - Tomato	SOBIR1/BAK1	Ave1	Hemibiotroph	Yes	Resistance
Cf-9 - Tomato	SOBIR1/BAK1	Avr9	Biotroph	Yes	Resistance
WAKs					
Stb6 – Wheat	None	AvrStb6	Necrotroph	No	Resistance
Snn1 – Wheat	None	Tox1	Necrotroph	Yes	Susceptibility
Rlm-9 – Canola	None	AvrLm5-9	Hemibiotroph	Yes	Resistance
WAK1 - *A. thaliana*	None	Oligogalacturonides	All	No	Resistance
Htn1 - Maize	None	Unknown	Hemibiotroph	No	Resistance
G-type lectin RLKs					
I-3 – Tomato	None	Avr3	Hemibiotroph	Yes	Resistance
NLRs					
LOV1 – *A. thaliana*	None	Victorin	Necrotroph	Yes	Susceptibility
Tsn1 – Wheat	None	ToxA	Necrotroph	Yes	Susceptibility
MSP-kinase					
Snn3 – Wheat	None	Tox3	Necrotroph	Yes	Susceptibility

This table is not an exhaustive list of all plant receptors that detect pathogenic fungi.

## Summary

- Pathogenic fungi use biotrophic, hemibiotrophic, and necrotrophic infection strategies to infect plants and reduce crop productivity.
- Plants produce a variety of receptor proteins that can detect danger from the cell-surface or inside the cell, leading to defense responses against diverse fungal pathogens.
- Recent evidence suggests that pathogenic fungi can interfere with receptor activation via the release of effectors with structural similarity to the recognized effectors.
- The success of an immune response can depend on the infection strategy of the invader. A hypersensitive cell-death response is often effective at preventing biotrophic growth but can be manipulated by necrotrophs to promote infection.

## Open Access

Open access for this article was enabled by the participation of Australian National University in an all-inclusive *Read & Publish* agreement with Portland Press and the Biochemical Society under a transformative agreement with CAUL.

## Abbreviations

DAMP: damage-associated molecular pattern
HR: hypersensitive response
LRR: leucine-rich repeat
MAMP: microbe-associated molecular pattern
MAPK: mitogen-activated protein kinase
NB: nucleotide binding
NLR: nucleotide binding leucine-rich repeat receptor
PAN: plasminogen-apple-nematode
ROS: reactive oxygen species
SLG: *S-*locus glycoprotein
WAK: Wall-associated kinase

## References

[1] Fisher M.C., Hawkins N.J., Sanglard D. and Gurr S.J. (2018) Worldwide emergence of resistance to antifungal drugs challenges human health and food security. Science 360, 739–742 10.1126/science.aap7999 29773744

[2] van der Burgh A.M. and Joosten M. (2019) Plant immunity: thinking outside and inside the box. Trends Plant Sci. 24, 587–601 10.1016/j.tplants.2019.04.009 31171472

[3] Kanyuka K. and Rudd J.J. (2019) Cell surface immune receptors: the guardians of the plant's extracellular spaces. Curr. Opin. Plant Biol. 50, 1–8 10.1016/j.pbi.2019.02.005 30861483 PMC6731392

[4] Lo Presti L., Lanver D., Schweizer G., Tanaka S., Liang L., Tollot M. et al. (2015) Fungal effectors and plant susceptibility. Annu. Rev. Plant Biol. 66, 513–545 10.1146/annurev-arplant-043014-114623 25923844

[5] Peng Y., van Wersch R. and Zhang Y. (2018) Convergent and divergent signaling in PAMP-triggered immunity and effector-triggered immunity. Mol. Plant. Microbe. Interact. 31, 403–409 10.1094/MPMI-06-17-0145-CR 29135338

[6] Tsuda K. and Katagiri F. (2010) Comparing signaling mechanisms engaged in pattern-triggered and effector-triggered immunity. Curr. Opin. Plant Biol. 13, 459–465 10.1016/j.pbi.2010.04.006 20471306

[7] Macho A.P. and Zipfel C. (2014) Plant PRRs and the activation of innate immune signaling. Mol. Cell. 54, 263–272 10.1016/j.molcel.2014.03.028 24766890

[8] Stephens C., Hammond-Kosack K.E. and Kanyuka K. (2022) WAKsing plant immunity, waning diseases. J. Exp. Bot. 73, 22–37 10.1093/jxb/erab422 34520537

[9] Miya A., Albert P., Shinya T., Desaki Y., Ichimura K., Shirasu K. et al. (2007) CERK1, a LysM receptor kinase, is essential for chitin elicitor signaling in Arabidopsis. Proc. Natl. Acad. Sci. U.S.A. 104, 19613–19618 10.1073/pnas.0705147104 18042724 PMC2148337

[10] Shimizu T., Nakano T., Takamizawa D., Desaki Y., Ishii-Minami N., Nishizawa Y. et al. (2010) Two LysM receptor molecules, CEBiP and OsCERK1, cooperatively regulate chitin elicitor signaling in rice. Plant J. 64, 204–214 10.1111/j.1365-313X.2010.04324.x 21070404 PMC2996852

[11] Hayafune M., Berisio R., Marchetti R., Silipo A., Kayama M., Desaki Y. et al. (2014) Chitin-induced activation of immune signaling by the rice receptor CEBiP relies on a unique sandwich-type dimerization. Proc. Natl. Acad Sci. U.S.A. 111, E404–E413 10.1073/pnas.1312099111 24395781 PMC3903257

[12] Miyata K., Kozaki T., Kouzai Y., Ozawa K., Ishii K., Asamizu E. et al. (2014) The bifunctional plant receptor, OsCERK1, regulates both chitin-triggered immunity and arbuscular mycorrhizal symbiosis in rice. Plant Cell Physiol. 55, 1864–1872 10.1093/pcp/pcu129 25231970

[13] Zhang X., Dong W., Sun J., Feng F., Deng Y., He Z. et al. (2015) The receptor kinase CERK1 has dual functions in symbiosis and immunity signalling. Plant J. 81, 258–267 10.1111/tpj.12723 25399831

[14] Carotenuto G., Chabaud M., Miyata K., Capozzi M., Takeda N., Kaku H. et al. (2017) The rice LysM receptor-like kinase OsCERK1 is required for the perception of short-chain chitin oligomers in arbuscular mycorrhizal signaling. New Phytol. 214, 1440–1446 10.1111/nph.14539 28369864

[15] He J., Zhang C., Dai H., Liu H., Zhang X., Yang J. et al. (2019) A LysM receptor heteromer mediates perception of arbuscular mycorrhizal symbiotic signal in rice. Mol. Plant 12, 1561–1576 10.1016/j.molp.2019.10.015 31706032

[16] Zhang C., He J., Dai H., Wang G., Zhang X., Wang C. et al. (2021) Discriminating symbiosis and immunity signals by receptor competition in rice. Proc. Natl. Acad Sci. U.S.A. 118, 10.1073/pnas.2023738118 PMC8072404 33853950

[17] Wan J., Tanaka K., Zhang X.C., Son G.H., Brechenmacher L., Nguyen T.H. et al. (2012) LYK4, a lysin motif receptor-like kinase, is important for chitin signaling and plant innate immunity in Arabidopsis. Plant Physiol. 160, 396–406 10.1104/pp.112.201699 22744984 PMC3440214

[18] Cao Y., Liang Y., Tanaka K., Nguyen C.T., Jedrzejczak R.P., Joachimiak A. et al. (2014) The kinase LYK5 is a major chitin receptor in Arabidopsis and forms a chitin-induced complex with related kinase CERK1. Elife 3, e03766, 10.7554/eLife.03766 PMC4356144 25340959

[19] Gong B.Q., Wang F.Z. and Li J.F. (2020) Hide-and-seek: chitin-triggered plant immunity and fungal counterstrategies. Trends Plant Sci. 25, 805–816 10.1016/j.tplants.2020.03.006 32673581

[20] Liu T., Liu Z., Song C., Hu Y., Han Z., She J. et al. (2012) Chitin-induced dimerization activates a plant immune receptor. Science 336, 1160–1164 10.1126/science.1218867 22654057

[21] Liu J., Liu B., Chen S., Gong B.Q., Chen L., Zhou Q. et al. (2018) A tyrosine phosphorylation cycle regulates fungal activation of a plant receptor Ser/Thr kinase. Cell Host Microbe 23, 241.e6–253.e6 10.1016/j.chom.2017.12.005 29396039

[22] Zhang J., Li W., Xiang T., Liu Z., Laluk K., Ding X. et al. (2010) Receptor-like cytoplasmic kinases integrate signaling from multiple plant immune receptors and are targeted by a Pseudomonas syringae effector. Cell Host Microbe 7, 290–301 10.1016/j.chom.2010.03.007 20413097

[23] Shinya T., Yamaguchi K., Desaki Y., Yamada K., Narisawa T., Kobayashi Y. et al. (2014) Selective regulation of the chitin-induced defense response by the Arabidopsis receptor-like cytoplasmic kinase PBL27. Plant J. 79, 56–66 10.1111/tpj.12535 24750441

[24] Bi G., Zhou Z., Wang W., Li L., Rao S., Wu Y. et al. (2018) Receptor-like cytoplasmic kinases directly link diverse pattern recognition receptors to the activation of mitogen-activated protein kinase cascades in Arabidopsis. Plant Cell. 30, 1543–1561 10.1105/tpc.17.00981 29871986 PMC6096590

[25] Yamada K., Yamaguchi K., Yoshimura S., Terauchi A. and Kawasaki T. (2017) Conservation of chitin-induced MAPK signaling pathways in rice and Arabidopsis. Plant Cell Physiol. 58, 993–1002 10.1093/pcp/pcx042 28371870

[26] Ao Y., Li Z., Feng D., Xiong F., Liu J., Li J.F. et al. (2014) OsCERK1 and OsRLCK176 play important roles in peptidoglycan and chitin signaling in rice innate immunity. Plant J. 80, 1072–1084 10.1111/tpj.12710 25335639

[27] Wang C., Wang G., Zhang C., Zhu P., Dai H., Yu N. et al. (2017) OsCERK1-mediated chitin perception and immune signaling requires receptor-like cytoplasmic kinase 185 to activate an MAPK cascade in Rice. Mol. Plant 10, 619–633 10.1016/j.molp.2017.01.006 28111288

[28] Ye W., Munemasa S., Shinya T., Wu W., Ma T., Lu J. et al. (2020) Stomatal immunity against fungal invasion comprises not only chitin-induced stomatal closure but also chitosan-induced guard cell death. Proc. Natl. Acad Sci. U.S.A. 117, 20932–20942 10.1073/pnas.1922319117 32778594 PMC7456093

[29] Liu Y., Maierhofer T., Rybak K., Sklenar J., Breakspear A., Johnston M.G. et al. (2019) Anion channel SLAH3 is a regulatory target of chitin receptor-associated kinase PBL27 in microbial stomatal closure. Elife 8, e44474, 10.7554/eLife.44474 31524595 PMC6776436

[30] Nolan T.M., Vukašinović N., Liu D., Russinova E. and Yin Y. (2020) Brassinosteroids: multidimensional regulators of plant growth, development, and stress responses. Plant Cell. 32, 295–318 10.1105/tpc.19.00335 31776234 PMC7008487

[31] Smakowska-Luzan E., Mott G.A., Parys K., Stegmann M., Howton T.C., Layeghifard M. et al. (2018) An extracellular network of Arabidopsis leucine-rich repeat receptor kinases. Nature 553, 342–346 10.1038/nature25184 29320478 PMC6485605

[32] Li J., Wen J., Lease K.A., Doke J.T., Tax F.E. and Walker J.C. (2002) BAK1, an Arabidopsis LRR receptor-like protein kinase, interacts with BRI1 and modulates brassinosteroid signaling. Cell 110, 213–222 10.1016/S0092-8674(02)00812-7 12150929

[33] Nam K.H. and Li J. (2002) BRI1/BAK1, a receptor kinase pair mediating brassinosteroid signaling. Cell 110, 203–212 10.1016/S0092-8674(02)00814-0 12150928

[34] Chinchilla D., Zipfel C., Robatzek S., Kemmerling B., Nürnberger T., Jones J.D. et al. (2007) A flagellin-induced complex of the receptor FLS2 and BAK1 initiates plant defence. Nature 448, 497–500 10.1038/nature05999 17625569

[35] Gómez-Gómez L. and Boller T. (2000) FLS2: an LRR receptor-like kinase involved in the perception of the bacterial elicitor flagellin in Arabidopsis. Mol. Cell. 5, 1003–1011 10.1016/S1097-2765(00)80265-8 10911994

[36] Li J. and Chory J. (1997) A putative leucine-rich repeat receptor kinase involved in brassinosteroid signal transduction. Cell 90, 929–938 10.1016/S0092-8674(00)80357-8 9298904

[37] Stegmann M., Monaghan J., Smakowska-Luzan E., Rovenich H., Lehner A., Holton N. et al. (2017) The receptor kinase FER is a RALF-regulated scaffold controlling plant immune signaling. Science 355, 287–289 10.1126/science.aal2541 28104890

[38] Jones D.A., Thomas C.M., Hammond-Kosack K.E., Balint-Kurti P.J. and Jones J.D. (1994) Isolation of the tomato Cf-9 gene for resistance to Cladosporium fulvum by transposon tagging. Science 266, 789–793 10.1126/science.7973631 7973631

[39] Dixon M.S., Hatzixanthis K., Jones D.A., Harrison K. and Jones J.D. (1998) The tomato Cf-5 disease resistance gene and six homologs show pronounced allelic variation in leucine-rich repeat copy number. Plant Cell. 10, 1915–1925 10.1105/tpc.10.11.1915 9811798 PMC143956

[40] Dixon M.S., Jones D.A., Keddie J.S., Thomas C.M., Harrison K. and Jones J.D. (1996) The tomato Cf-2 disease resistance locus comprises two functional genes encoding leucine-rich repeat proteins. Cell 84, 451–459 10.1016/S0092-8674(00)81290-8 8608599

[41] Thomas C.M., Jones D.A., Parniske M., Harrison K., Balint-Kurti P.J., Hatzixanthis K. et al. (1997) Characterization of the tomato Cf-4 gene for resistance to Cladosporium fulvum identifies sequences that determine recognitional specificity in Cf-4 and Cf-9. Plant Cell. 9, 2209–2224 9437864 10.1105/tpc.9.12.2209 PMC157069

[42] Takken F.L., Thomas C.M., Joosten M.H., Golstein C., Westerink N., Hille J. et al. (1999) A second gene at the tomato Cf-4 locus confers resistance to cladosporium fulvum through recognition of a novel avirulence determinant. Plant J. 20, 279–288 10.1046/j.1365-313X.1999.t01-1-00601.x 10571888

[43] Liebrand T.W., van den Berg G.C., Zhang Z., Smit P., Cordewener J.H., America A.H. et al. (2013) Receptor-like kinase SOBIR1/EVR interacts with receptor-like proteins in plant immunity against fungal infection. Proc. Natl. Acad Sci. U.S.A. 110, 10010–10015 10.1073/pnas.1220015110 23716655 PMC3683720

[44] Postma J., Liebrand T.W., Bi G., Evrard A., Bye R.R., Mbengue M. et al. (2016) Avr4 promotes Cf-4 receptor-like protein association with the BAK1/SERK3 receptor-like kinase to initiate receptor endocytosis and plant immunity. New Phytol. 210, 627–642 10.1111/nph.13802 26765243

[45] Cai X., Takken F.L., Joosten M.H. and De Wit P.J. (2001) Specific recognition of AVR4 and AVR9 results in distinct patterns of hypersensitive cell death in tomato, but similar patterns of defence-related gene expression. Mol. Plant Pathol. 2, 77–86 10.1046/j.1364-3703.2001.00053.x 20572994

[46] de Jonge R., van Esse H.P., Maruthachalam K., Bolton M.D., Santhanam P., Saber M.K. et al. (2012) Tomato immune receptor Ve1 recognizes effector of multiple fungal pathogens uncovered by genome and RNA sequencing. Proc. Natl. Acad Sci. U.S.A. 109, 5110–5115 10.1073/pnas.1119623109 22416119 PMC3323992

[47] Kawchuk L.M., Hachey J., Lynch D.R., Kulcsar F., van Rooijen G., Waterer D.R. et al. (2001) Tomato Ve disease resistance genes encode cell surface-like receptors. Proc. Natl. Acad Sci. U.S.A. 98, 6511–6515 10.1073/pnas.091114198 11331751 PMC33499

[48] Fradin E.F., Zhang Z., Juarez Ayala J.C., Castroverde C.D., Nazar R.N., Robb J. et al. (2009) Genetic dissection of Verticillium wilt resistance mediated by tomato Ve1. Plant Physiol. 150, 320–332 10.1104/pp.109.136762 19321708 PMC2675724

[49] Zhang W., Fraiture M., Kolb D., Löffelhardt B., Desaki Y., Boutrot F.F. et al. (2013) Arabidopsis receptor-like protein30 and receptor-like kinase suppressor of BIR1-1/EVERSHED mediate innate immunity to necrotrophic fungi. Plant Cell. 25, 4227–4241 10.1105/tpc.113.117010 24104566 PMC3877809

[50] Böhm H., Albert I., Oome S., Raaymakers T.M., Van den Ackerveken G. and Nürnberger T. (2014) A conserved peptide pattern from a widespread microbial virulence factor triggers pattern-induced immunity in Arabidopsis. PLoS Pathog. 10, e1004491 10.1371/journal.ppat.1004491 25375108 PMC4223075

[51] Albert I., Böhm H., Albert M., Feiler C.E., Imkampe J., Wallmeroth N. et al. (2015) An RLP23-SOBIR1-BAK1 complex mediates NLP-triggered immunity. Nat. Plant. 1, 15140 10.1038/nplants.2015.140 27251392

[52] Lenarčič T., Albert I., Böhm H., Hodnik V., Pirc K., Zavec A.B. et al. (2017) Eudicot plant-specific sphingolipids determine host selectivity of microbial NLP cytolysins. Science 358, 1431–1434 10.1126/science.aan6874 29242345

[53] Chen J.B., Bao S.W., Fang Y.L., Wei L.Y., Zhu W.S., Peng Y.L. et al. (2021) An LRR-only protein promotes NLP-triggered cell death and disease susceptibility by facilitating oligomerization of NLP in Arabidopsis. New Phytol. 232, 1808–1822 10.1111/nph.17680 34403491

[54] Kleemann J., Rincon-Rivera L.J., Takahara H., Neumann U., Ver Loren van Themaat E., van der Does H.C. et al. (2012) Sequential delivery of host-induced virulence effectors by appressoria and intracellular hyphae of the phytopathogen Colletotrichum higginsianum. PLoS Pathog. 8, e1002643 10.1371/journal.ppat.1002643 22496661 PMC3320591

[55] Gijzen M. and Nürnberger T. (2006) Nep1-like proteins from plant pathogens: recruitment and diversification of the NPP1 domain across taxa. Phytochemistry 67, 1800–1807 10.1016/j.phytochem.2005.12.008 16430931

[56] Ono E., Mise K. and Takano Y. (2020) RLP23 is required for Arabidopsis immunity against the grey mould pathogen Botrytis cinerea. Sci. Rep. 10, 13798 10.1038/s41598-020-70485-1 32796867 PMC7428006

[57] Boutrot F. and Zipfel C. (2017) Function, discovery, and exploitation of plant pattern recognition receptors for broad-spectrum disease resistance. Annu. Rev. Phytopathol. 55, 257–286 10.1146/annurev-phyto-080614-120106 28617654

[58] Wu J., Reca I.B., Spinelli F., Lironi D., De Lorenzo G., Poltronieri P. et al. (2019) An EFR-Cf-9 chimera confers enhanced resistance to bacterial pathogens by SOBIR1- and BAK1-dependent recognition of elf18. Mol. Plant Pathol. 20, 751–764 10.1111/mpp.12789 30938041 PMC6637901

[59] Perraki A., DeFalco T.A., Derbyshire P., Avila J., Séré D., Sklenar J. et al. (2018) Phosphocode-dependent functional dichotomy of a common co-receptor in plant signalling. Nature 561, 248–252 10.1038/s41586-018-0471-x 30177827 PMC6250601

[60] Sun Y., Qiao Z., Muchero W. and Chen J.G. (2020) Lectin receptor-like kinases: the sensor and mediator at the plant cell surface. Front Plant Sci. 11, 596301 10.3389/fpls.2020.596301 33362827 PMC7758398

[61] Bellande K., Bono J.J., Savelli B., Jamet E. and Canut H. (2017) Plant lectins and lectin receptor-like kinases: how do they sense the outside? Int. J. Mol. Sci. 18, 10.3390/ijms18061164 28561754 PMC5485988

[62] Sherman-Broyles S., Boggs N., Farkas A., Liu P., Vrebalov J., Nasrallah M.E. et al. (2007) S locus genes and the evolution of self-fertility in Arabidopsis thaliana. Plant Cell. 19, 94–106 10.1105/tpc.106.048199 17237349 PMC1820967

[63] Kusaba M., Dwyer K., Hendershot J., Vrebalov J., Nasrallah J.B. and Nasrallah M.E. (2001) Self-incompatibility in the genus Arabidopsis: characterization of the S locus in the outcrossing A. lyrata and its autogamous relative A. thaliana. Plant Cell 13, 627–643 10.1105/tpc.13.3.627 11251101 PMC135518

[64] Teixeira M.A., Rajewski A., He J., Castaneda O.G., Litt A. and Kaloshian I. (2018) Classification and phylogenetic analyses of the Arabidopsis and tomato G-type lectin receptor kinases. BMC Genomics 19, 239 10.1186/s12864-018-4606-0 29625550 PMC5889549

[65] Kemp B.P. and Doughty J. (2007) S cysteine-rich (SCR) binding domain analysis of the Brassica self-incompatibility S-locus receptor kinase. New Phytol. 175, 619–629 10.1111/j.1469-8137.2007.02126.x 17688579

[66] Ma R., Han Z., Hu Z., Lin G., Gong X., Zhang H. et al. (2016) Structural basis for specific self-incompatibility response in Brassica. Cell Res. 26, 1320–1329 10.1038/cr.2016.129 27824028 PMC5143417

[67] Giranton J.L., Dumas C., Cock J.M. and Gaude T. (2000) The integral membrane S-locus receptor kinase of Brassica has serine/threonine kinase activity in a membranous environment and spontaneously forms oligomers in planta. Proc. Natl. Acad Sci. U.S.A. 97, 3759–3764 10.1073/pnas.97.7.3759 10725390 PMC16313

[68] Wang L., Lin Z., Triviño M., Nowack M.K., Franklin-Tong V.E. and Bosch M. (2019) Self-incompatibility in Papaver pollen: programmed cell death in an acidic environment. J. Exp. Bot. 70, 2113–2123 30481323 10.1093/jxb/ery406 PMC7116307

[69] Haque T., Eaves D.J., Lin Z., Zampronio C.G., Cooper H.J., Bosch M. et al. (2020) Self-Incompatibility Triggers Irreversible Oxidative Modification of Proteins in Incompatible Pollen. Plant Physiol. 183, 1391–1404 10.1104/pp.20.00066 32321844 PMC7333688

[70] Franklin-Tong V.E., Ride J.P., Read N.D., Trewavas A.J. and Franklin F.C.H. (1993) The self-incompatibility response in Papaver rhoeas is mediated by cytosolic free calcium. Plant J. 4, 163–177 10.1046/j.1365-313X.1993.04010163.x

[71] Wilkins K.A., Bosch M., Haque T., Teng N., Poulter N.S. and Franklin-Tong V.E. (2015) Self-incompatibility-induced programmed cell death in field poppy pollen involves dramatic acidification of the incompatible pollen tube cytosol. Plant Physiol. 167, 766–779 10.1104/pp.114.252742 25630437 PMC4347735

[72] Wilkins K.A., Bancroft J., Bosch M., Ings J., Smirnoff N. and Franklin-Tong V.E. (2011) Reactive oxygen species and nitric oxide mediate actin reorganization and programmed cell death in the self-incompatibility response of papaver. Plant Physiol. 156, 404–416 10.1104/pp.110.167510 21386034 PMC3091060

[73] Thomas S.G. and Franklin-Tong V.E. (2004) Self-incompatibility triggers programmed cell death in Papaver pollen. Nature 429, 305–309 10.1038/nature02540 15152254

[74] Catanzariti A.M., Lim G.T.T. and Jones D.A. (2015) The tomato I-3 gene: a novel gene for resistance to Fusarium wilt disease. New Phytol. 207, 106–118 10.1111/nph.13348 25740416

[75] Yu D.S., Outram M.A., Smith A., McCombe C.L., Khambalkar P.B., Rima S.A. et al. (2021) The structural repertoire of *Fusarium oxysporum* f. sp. *lycopersici* effectors revealed by experimental and computational studies. bioRxiv, 2021.12.14.472499, 10.1016/2021.12.14.472499 PMC10942635 38411527

[76] Rep M., van der Does H.C., Meijer M., van Wijk R., Houterman P.M., Dekker H.L. et al. (2004) A small, cysteine-rich protein secreted by Fusarium oxysporum during colonization of xylem vessels is required for I-3-mediated resistance in tomato. Mol. Microbiol. 53, 1373–1383 10.1111/j.1365-2958.2004.04177.x 15387816

[77] Catanzariti A.M., Do H.T., Bru P., de Sain M., Thatcher L.F., Rep M. et al. (2017) The tomato I gene for Fusarium wilt resistance encodes an atypical leucine-rich repeat receptor-like protein whose function is nevertheless dependent on SOBIR1 and SERK3/BAK1. Plant J. 89, 1195–1209 10.1111/tpj.13458 27995670

[78] Houterman P.M., Cornelissen B.J. and Rep M. (2008) Suppression of plant resistance gene-based immunity by a fungal effector. PLoS Pathog. 4, e1000061 10.1371/journal.ppat.1000061 18464895 PMC2330162

[79] Verica J.A. and He Z.H. (2002) The cell wall-associated kinase (WAK) and WAK-like kinase gene family. Plant Physiol. 129, 455–459 10.1104/pp.011028 12068092 PMC1540232

[80] Zhang Z., Ma W., Ren Z., Wang X., Zhao J., Pei X. et al. (2021) Characterization and expression analysis of wall-associated kinase (WAK) and WAK-like family in cotton. Int. J. Biol. Macromol. 187, 867–879 10.1016/j.ijbiomac.2021.07.163 34339786

[81] Tripathi R.K., Aguirre J.A. and Singh J. (2021) Genome-wide analysis of wall associated kinase (WAK) gene family in barley. Genomics 113, 523–530 10.1016/j.ygeno.2020.09.045 32987151

[82] Zhang S., Chen C., Li L., Meng L., Singh J., Jiang N. et al. (2005) Evolutionary expansion, gene structure, and expression of the rice wall-associated kinase gene family. Plant Physiol. 139, 1107–1124 10.1104/pp.105.069005 16286450 PMC1283751

[83] Appels R., Eversole K., Feuillet C., Keller B., Rogers J., Stein N. et al. (2018) Shifting the limits in wheat research and breeding using a fully annotated reference genome. Science 361, 6403, eaar7191, 10.1126/science.aar7191 30115783

[84] He Z.H., Fujiki M. and Kohorn B.D. (1996) A cell wall-associated, receptor-like protein kinase. J. Biol. Chem. 271, 19789–19793 10.1074/jbc.271.33.19789 8702686

[85] Wagner T.A. and Kohorn B.D. (2001) Wall-associated kinases are expressed throughout plant development and are required for cell expansion. Plant Cell. 13, 303–318 10.1105/tpc.13.2.303 11226187 PMC102244

[86] Decreux A. and Messiaen J. (2005) Wall-associated kinase WAK1 interacts with cell wall pectins in a calcium-induced conformation. Plant Cell Physiol. 46, 268–278 10.1093/pcp/pci026 15769808

[87] Brutus A., Sicilia F., Macone A., Cervone F. and De Lorenzo G. (2010) A domain swap approach reveals a role of the plant wall-associated kinase 1 (WAK1) as a receptor of oligogalacturonides. Proc. Natl. Acad Sci. U.S.A. 107, 9452–9457 10.1073/pnas.1000675107 20439716 PMC2889104

[88] De Lorenzo G., Brutus A., Savatin D.V., Sicilia F. and Cervone F. (2011) Engineering plant resistance by constructing chimeric receptors that recognize damage-associated molecular patterns (DAMPs). FEBS Lett. 585, 1521–1528 10.1016/j.febslet.2011.04.043 21536040

[89] Saintenac C., Lee W.S., Cambon F., Rudd J.J., King R.C., Marande W. et al. (2018) Wheat receptor-kinase-like protein Stb6 controls gene-for-gene resistance to fungal pathogen Zymoseptoria tritici. Nat. Genet. 50, 368–374 10.1038/s41588-018-0051-x 29434355

[90] Larkan N.J., Ma L., Haddadi P., Buchwaldt M., Parkin I.A.P., Djavaheri M. et al. (2020) The Brassica napus wall-associated kinase-like (WAKL) gene Rlm9 provides race-specific blackleg resistance. Plant J. 104, 892–900 10.1111/tpj.14966 32794614 PMC7756564

[91] Haddadi P., Larkan N.J., Van de Wouw A., Zhang Y., Neik T.X., Beynon E. et al. (2021) *Brassica napus* genes *Rlm4* and *Rlm7,* conferring resistance to *Leptosphaeria maculans,* are alleles of the *Rlm9* wall-associated kinase-like resistance locus. bioRxiv, 2021.12.11.471845, 10.1101/2021.12.11.471845 PMC9241367 35338565

[92] Lazar N., Mesarich C.H., Petit-Houdenot Y., Talbi N., de la Sierra-Gallay I.L., Zélie E. et al. (2021) A new family of structurally conserved fungal effectors displays epistatic interactions with plant resistance proteins. bioRxiv, 2020.12.17.423041, 10.1101/2020.12.17.423041 PMC9292093 35793393

[93] Ghanbarnia K., Ma L., Larkan N.J., Haddadi P., Fernando W.G.D. and Borhan M.H. (2018) Leptosphaeria maculans AvrLm9: a new player in the game of hide and seek with AvrLm4-7. Mol. Plant Pathol. 19, 1754–1764 10.1111/mpp.12658 29330918 PMC6638032

[94] Shi G., Zhang Z., Friesen T.L., Raats D., Fahima T., Brueggeman R.S. et al. (2016) The hijacking of a receptor kinase-driven pathway by a wheat fungal pathogen leads to disease. Sci. Adv. 2, e1600822 10.1126/sciadv.1600822 27819043 PMC5091353

[95] Liu Z., Zhang Z., Faris J.D., Oliver R.P., Syme R., McDonald M.C. et al. (2012) The cysteine rich necrotrophic effector SnTox1 produced by Stagonospora nodorum triggers susceptibility of wheat lines harboring Snn1. PLoS Pathog. 8, e1002467 10.1371/journal.ppat.1002467 22241993 PMC3252377

[96] McDonald M.C. and Solomon P.S. (2018) Just the surface: advances in the discovery and characterization of necrotrophic wheat effectors. Curr. Opin. Microbiol. 46, 14–18 10.1016/j.mib.2018.01.019 29452845

[97] Hurni S., Scheuermann D., Krattinger S.G., Kessel B., Wicker T., Herren G. et al. (2015) The maize disease resistance gene Htn1 against northern corn leaf blight encodes a wall-associated receptor-like kinase. Proc. Natl. Acad Sci. U.S.A. 112, 8780–8785 10.1073/pnas.1502522112 26124097 PMC4507197

[98] Yang P., Scheuermann D., Kessel B., Koller T., Greenwood J.R., Hurni S. et al. (2021) Alleles of a wall-associated kinase gene account for three of the major northern corn leaf blight resistance loci in maize. Plant J. 106, 526–535 10.1111/tpj.15183 33533097

[99] Yang P., Praz C., Li B., Singla J., Robert C.A.M., Kessel B. et al. (2019) Fungal resistance mediated by maize wall-associated kinase ZmWAK-RLK1 correlates with reduced benzoxazinoid content. New Phytol. 221, 976–987 10.1111/nph.15419 30178602

[100] Rui Y. and Dinneny J.R. (2020) A wall with integrity: surveillance and maintenance of the plant cell wall under stress. New Phytol. 225, 1428–1439 10.1111/nph.16166 31486535

[101] Hu W., Lv Y., Lei W., Li X., Chen Y., Zheng L. et al. (2014) Cloning and characterization of the Oryza sativa wall-associated kinase gene OsWAK11 and its transcriptional response to abiotic stresses. Plant Soil 384, 335–346 10.1007/s11104-014-2204-8

[102] Balint-Kurti P. (2019) The plant hypersensitive response: concepts, control and consequences. Mol. Plant Pathol. 20, 1163–1178 31305008 10.1111/mpp.12821 PMC6640183

[103] Jubic L.M., Saile S., Furzer O.J., El Kasmi F. and Dangl J.L. (2019) Help wanted: helper NLRs and plant immune responses. Curr. Opin. Plant Biol. 50, 82–94 10.1016/j.pbi.2019.03.013 31063902

[104] Bentham A.R., De la Concepcion J.C., Mukhi N., Zdrzałek R., Draeger M., Gorenkin D. et al. (2020) A molecular roadmap to the plant immune system. J. Biol. Chem. 295, 14916–14935 10.1074/jbc.REV120.010852 32816993 PMC7606695

[105] van Wersch S., Tian L., Hoy R. and Li X. (2020) Plant NLRs: the whistleblowers of plant immunity. Plant Commun. 1, 100016 10.1016/j.xplc.2019.100016 33404540 PMC7747998

[106] Zhou J.M. and Zhang Y. (2020) Plant immunity: danger perception and signaling. Cell 181, 978–989 10.1016/j.cell.2020.04.028 32442407

[107] Lorang J.M., Sweat T.A. and Wolpert T.J. (2007) Plant disease susceptibility conferred by a “resistance” gene. Proc. Natl. Acad Sci. U.S.A. 104, 14861–14866 10.1073/pnas.0702572104 17804803 PMC1976202

[108] Lorang J., Kidarsa T., Bradford C.S., Gilbert B., Curtis M., Tzeng S.C. et al. (2012) Tricking the guard: exploiting plant defense for disease susceptibility. Science 338, 659–662 10.1126/science.1226743 23087001 PMC4125361

[109] Kessler S.C., Zhang X., McDonald M.C., Gilchrist C.L.M., Lin Z., Rightmyer A. et al. (2020) Victorin, the host-selective cyclic peptide toxin from the oat pathogen Cochliobolus victoriae, is ribosomally encoded. Proc. Natl. Acad Sci. U.S.A. 117, 24243–24250 10.1073/pnas.2010573117 32929037 PMC7533671

[110] Sweat T.A., Lorang J.M., Bakker E.G. and Wolpert T.J. (2008) Characterization of natural and induced variation in the LOV1 gene, a CC-NB-LRR gene conferring victorin sensitivity and disease susceptibility in Arabidopsis. Mol. Plant. Microbe. Interact. 21, 7–19 10.1094/MPMI-21-1-0007 18052878

[111] Litzenberger S. (1949) Nature of susceptibility to Helminthosporium victoriae and resistance to Puccinia coronata in Victoria oats. Phytopathology 39, 300–318

[112] Wolpert T.J. and Lorang J.M. (2016) Victoria Blight, defense turned upside down. Physiol. Mol. Pl. Path. 95, 8–13 10.1016/j.pmpp.2016.03.006

[113] Faris J.D., Zhang Z., Lu H., Lu S., Reddy L., Cloutier S. et al. (2010) A unique wheat disease resistance-like gene governs effector-triggered susceptibility to necrotrophic pathogens. Proc. Natl. Acad Sci. U.S.A. 107, 13544–13549 10.1073/pnas.1004090107 20624958 PMC2922177

[114] Tuori R.P., Wolpert T.J. and Ciuffetti L.M. (1995) Purification and immunological characterization of toxic components from cultures of Pyrenophora tritici-repentis. Mol. Plant. Microbe. Interact. 8, 41–48 10.1094/MPMI-8-0041 7772802

[115] Friesen T.L., Holmes D.J., Bowden R.L. and Faris J.D. (2018) ToxA is present in the U.S. bipolaris sorokiniana population and is a significant virulence factor on wheat harboring Tsn1. Plant Dis. 102, 2446–2452 10.1094/PDIS-03-18-0521-RE 30252627

[116] Ciuffetti L.M., Tuori R.P. and Gaventa J.M. (1997) A single gene encodes a selective toxin causal to the development of tan spot of wheat. Plant Cell. 9, 135–144 9061946 10.1105/tpc.9.2.135 PMC156906

[117] McDonald M.C., Ahren D., Simpfendorfer S., Milgate A. and Solomon P.S. (2018) The discovery of the virulence gene ToxA in the wheat and barley pathogen Bipolaris sorokiniana. Mol. Plant Pathol. 19, 432–439 10.1111/mpp.12535 28093843 PMC6638140

[118] Friesen T.L., Stukenbrock E.H., Liu Z., Meinhardt S., Ling H., Faris J.D. et al. (2006) Emergence of a new disease as a result of interspecific virulence gene transfer. Nat. Genet. 38, 953–956 10.1038/ng1839 16832356

[119] Zhang Z., Running K.L.D., Seneviratne S., Peters Haugrud A.R., Szabo-Hever A., Shi G. et al. (2021) A protein kinase-major sperm protein gene hijacked by a necrotrophic fungal pathogen triggers disease susceptibility in wheat. Plant J. 106, 720–732 10.1111/tpj.15194 33576059

[120] Winterberg B., Du Fall L.A., Song X., Pascovici D., Care N., Molloy M. et al. (2014) The necrotrophic effector protein SnTox3 re-programs metabolism and elicits a strong defence response in susceptible wheat leaves. BMC Plant Biol. 14, 215 10.1186/s12870-014-0215-5 25123935 PMC4243954

[121] Faris J.D. and Friesen T.L. (2020) Plant genes hijacked by necrotrophic fungal pathogens. Curr. Opin. Plant Biol. 56, 74–80 10.1016/j.pbi.2020.04.003 32492572

[122] Gong B.Q., Guo J., Zhang N., Yao X., Wang H.B. and Li J.F. (2019) Cross-microbial protection via priming a conserved immune co-receptor through juxtamembrane phosphorylation in plants. Cell Host Microbe 26, 810.e7–822.e7 10.1016/j.chom.2019.10.010 31830443

[123] Wang P., Zhou L., Jamieson P., Zhang L., Zhao Z., Babilonia K. et al. (2020) The cotton wall-associated kinase GhWAK7A mediates responses to fungal wilt pathogens by complexing with the chitin sensory receptors. Plant Cell. 32, 3978–4001 10.1105/tpc.19.00950 33037150 PMC7721343

[124] Zhang N., Pombo M.A., Rosli H.G. and Martin G.B. (2020) Tomato wall-associated kinase SlWak1 depends on Fls2/Fls3 to promote apoplastic immune responses to pseudomonas syringae. Plant Physiol. 183, 1869–1882 10.1104/pp.20.00144 32371523 PMC7401122

[125] Kourelis J., Contreras M.P., Harant A., Adachi H., Derevnina L., Wu C.-H. et al. (2021) The helper NLR immune protein NRC3 mediates the hypersensitive cell death caused by the cell-surface receptor Cf-4. bioRxiv, 2021.09.28.461843, 10.1101/2021.09.28.461843 PMC9543701 36137148

[126] Gabriëls S.H., Vossen J.H., Ekengren S.K., van Ooijen G., Abd-El-Haliem A.M., van den Berg G.C. et al. (2007) An NB-LRR protein required for HR signalling mediated by both extra- and intracellular resistance proteins. Plant J. 50, 14–28 10.1111/j.1365-313X.2007.03027.x 17346268

[127] Yuan M., Jiang Z., Bi G., Nomura K., Liu M., Wang Y. et al. (2021) Pattern-recognition receptors are required for NLR-mediated plant immunity. Nature 592, 105–109 33692546 10.1038/s41586-021-03316-6 PMC8016741

[128] Ngou B.P.M., Ahn H.K., Ding P. and Jones J.D.G. (2021) Mutual potentiation of plant immunity by cell-surface and intracellular receptors. Nature 592, 110–115 10.1038/s41586-021-03315-7 33692545

[129] Yuan M., Ngou B.P.M., Ding P. and Xin X.F. (2021) PTI-ETI crosstalk: an integrative view of plant immunity. Curr. Opin. Plant Biol. 62, 102030 10.1016/j.pbi.2021.102030 33684883

[130] Ngou B.P.M., Jones J.D.G. and Ding P. (2021) Plant immune networks. Trends Plant Sci., 27, 255–273 34548213 10.1016/j.tplants.2021.08.012

[131] Lu Y. and Tsuda K. (2021) Intimate association of PRR- and NLR-mediated signaling in plant immunity. Mol. Plant. Microbe. Interact. 34, 3–14 10.1094/MPMI-08-20-0239-IA 33048599

